# Concerted Halogen Bonding and Orthogonal Metal-Halogen Interactions in Dimers of Lithium Formamidinate and Halogenated Formamidines: An *ab Initio* Study

**DOI:** 10.3390/molecules19011069

**Published:** 2014-01-17

**Authors:** Ruben D. Parra

**Affiliations:** Department of Chemistry, DePaul University 1110 W. Belden Ave., Chicago, IL 60614, USA; E-Mail: rparra1@depaul.edu; Tel.: +1-773-325-4343

**Keywords:** halogen bonding, orthogonal self-assembling interactions, QTAIM, *ab initio*

## Abstract

Dimers of lithium formamidinate, CH(NH)_2_Li, and halogenated formamidines, HN=CHNHX, (X=Cl, Br, or I) are used as model systems to investigate simultaneous N-X···N and N-Li···N interactions, in tandem with orthogonal Li···X interactions. Geometry optimizations and energy calculations for the dimers are examined with the MP2 method and the M06-2X hybrid functional and the aug-cc-pVTZ basis set (the aug-cc-pVTZ-PP basis set is used for the iodine atom). Both methods predict the formation of a planar structure of C_2v_ symmetry, regardless of the identity of the halogen atom. In this structure, the identities of the constituent monomers are essentially lost. Accordingly, the N-X···N interactions emerge as a rather symmetric quasi-linear N···X···N, where the covalent N-X bond in the halogenated formamidine is replaced by a partly covalent N···X interaction. Formation of the C_2v_ structure is also driven by a fairly linear N···Li···N interaction parallel to the N···X···N interaction, and a Li···X interaction orthogonal to both the N···X···N and N···Li···N interactions. The strength of the interactions increases with the size of the halogen. The robustness of the interactions suggests that the dimers studied here or suitable analogues may find diverse applications including their use as novel polymeric synthons.

## 1. Introduction

A halogen bond (XB) interaction is commonly understood as an attractive and highly directional interaction Y···X-R between an electron rich species or Lewis base, Y, and the halogen atom, X, from a molecule or fragment R–X in which R is a group more electronegative than X, or is X itself [1,2,3,4,5]. Very recently, the International Union of Pure and Applied Chemistry (IUPAC) released the following recommended definition for a halogen bond: a halogen bond occurs when there is evidence of a net attractive interaction between an electrophilic region associated with a halogen atom in a molecular entity and a nucleophilic region in another, or the same, molecular entity [6]. The strength and directionality of the XB interaction have been rationalized in terms of the so called σ-hole, which is the positive electrostatic potential that a covalently bonded halogen atom may develop in its outer side, opposite to the covalent bond and pointing toward any potential electron donor [7,8]. Because a halogen bond can form even in the absence of a σ hole, it has been proposed more generally that a halogen bond results from polarization of charge density on the acceptor and donor moieties. Polarization results in regions of charge depletion and charge concentration that complement each other giving rise to the attractive interaction known as the XB interaction. Such bonding model is known as the lump-hole theory, and shows that a true positive permanent σ-hole is not mandatory for XB formation [9]. The strength of the halogen bond is known to increase with the size (or polarizability) of the halogen atom, and for the same halogen, to increase with increasing electron-withdrawing ability of the atom or group of atoms to which the halogen atom is covalently bonded. Theoretical analysis and benchmarking of halogen bonds have been the subject of some fairly recent publications [10,11]. It has been demonstrated that halogen bonds should be studied with large basis sets and theoretical models that efficiently incorporate electrostatic, dispersion, polarization, and charge transfer interaction components.

The XB interaction is currently an important research focus of the scientific community at large, and in fact it has been so especially over the last two decades. The recent publication of a book and a number of general and specialized review articles attest to the growing interest on the nature of the halogen bond, and on its systematic use as a means of expanding the horizons of chemistry [12,13,14,15,16,17,18,19]. For example, the applications of halogen bonding in medicine and chemical biology are being intensely pursued by many groups; efforts in this direction include the development of force fields that account for the XB interactions in molecular docking programs used for lead optimization and hit identification in the drug design process. Likewise, applications of the halogen bond in synthetic chemistry, crystal engineering, and materials science have been widely documented. Because the halogen bond interaction shares similarities with the more familiar hydrogen bond (HB) interaction, many researchers have conducted systematic studies to compare their relative strengths, as well as the extent of cooperative effects in chains of XB or HB interactions [20,21,22,23,24,25,26,27,28,29]. These studies demonstrate that the halogen bond and the hydrogen bond could compete and even interfere with each other, but the two interactions could also coexist and cooperate in building more complex and functional structures. Additionally, there have been reports that the strength of a halogen bond can be strong enough to result in a partly covalent interaction. Particularly interesting are the recent reports of experimental evidence confirming the existence of symmetric [N···X···N]^+^ (X = I or Br) interactions both in solution and in the crystal [30,31]. Similarly, a computational study on dimers and trimers of halogenated formamidines shows that the iodine bond interaction, N-I···N, in the trimer becomes so strong that it results in the iodine atom being symmetrically positioned between the two nitrogen atoms, N···I···N [32]. Moreover, the bonding nature of the halonium ions in dipyridine complex [pyr-X-Pyr]^+^ (X = F, Cl, Br, I) was also very recently assessed using both theoretical and synthetic techniques [33]. It was found that iodine behaves as a classical Lewis acid resulting in a coordination complex, whereas the bonding nature of the chlorine and bromine was mostly on the covalent side, and that of the fluorine on the ion-molecule interaction side.

**Scheme 1:**
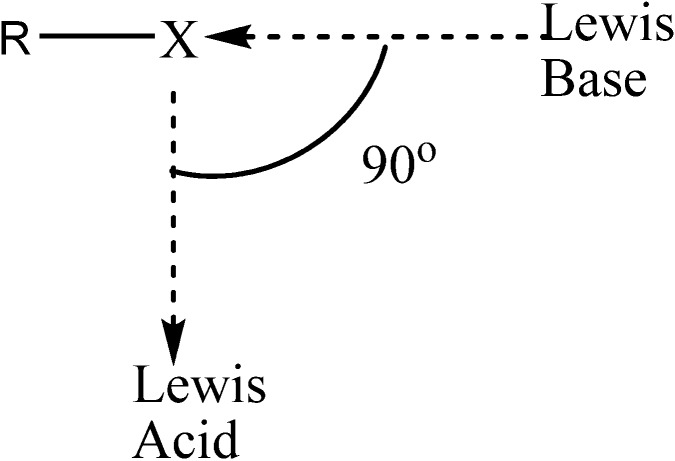
Graphical representation of the ability of a covalently bonded halogen atom to receive electron density from a Lewis base, and simultaneously provide perpendicularly electron density to a Lewis acid.

One distinctive characteristic of the halogen bond is the capacity that the halogen atom has to interact with an electron deficient species in a direction orthogonal to that of the halogen bond itself. Thus as illustrated in the Scheme 1, a halogen atom exhibits an amphoteric character which enables it to act as an electrophile (Lewis acid) in one direction, and as a nucleophile (Lewis base) in an orthogonal direction [34,35,36,37].

Along this line, the primary purpose of the present study is to contribute to the growing overall understanding and potential application of the halogen bond. In particular, the amphoteric character of a covalently bound halogen atom is examined using lithium formamidinate and halogenated formamidine as model systems for dimer interactions. Here the idea is to exploit the dual role that a halogen atom in the N-X···N interaction can play: in one role, the halogen atom is involved in a halogen bonding interaction through its σ-hole; in the other role, the halogen atom can interact with the lithium atom (electron-deficient species) in the formamidinate unit, through the negative electrostatic potential belt in the halogen atom that surrounds its σ-hole.

## 2. Computational Details

Geometry optimizations, frequency calculations, and single-point energy calculations were carried out using the GAUSSIAN 09 program [38]. Both the M06-2X hybrid functional and the MP2 method along with the aug-cc-pVDZ, or aug-cc-pVTZ basis sets were used to optimize the geometries of the dimers of lithium formamidinate, CH(NH)_2_Li, and halogenated formamidines, HN=CHNHX, (X=Cl, Br, or I) [39,40]. To account for relativistic effects, the aug-cc-pVDZ-PP or aug-cc-pVTZ-PP basis set was used for the iodine atom [41]. Frequency calculations carried out using the smaller basis set confirmed that the optimized structures were indeed minima in their respective potential energy surfaces. Interaction energies were corrected for basis set superposition error (BSSE) using the Boys-Bernardi counterpoise procedure [42]. The theory of atoms in molecules (AIM) of Bader [43] as implemented in the AIM2000, and AIMALL software packages [44,45] was employed to analyze topological features of electron density of the optimized structures. AIM analyses were performed on B3LYP density functional [46] and the aug-cc-pVTZ wavefunctions of minimum energy structures obtained with the same basis set and either the MP2 method or the M06-2X functional. Again, for the systems containing iodine atoms the aug-cc-pVTZ-PP basis set for I, and aug-cc-pVTZ basis set for the other atoms were used for the AIM calculations.

## 3. Results and Discussion

### 3.1. Geometries

**Figure 1:**
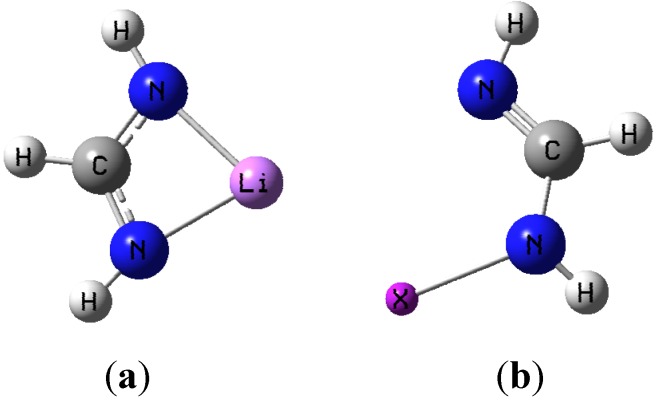
(**a**) Lithium formamidinate; (**b**) Halogenated formamidine (X = Cl, Br, or I).

Geometries obtained with both aug-cc-pVDZ and aug-cc-pVTZ basis sets (with corresponding aug-cc-pVDZ-PP and aug-cc-pVTZ-PP for iodine) are similar, and thus only the results with the larger basis set will be used for discussion. Figure 1a,b show the general shape of the two monomer structures used as basic motifs.

**Figure 2:**
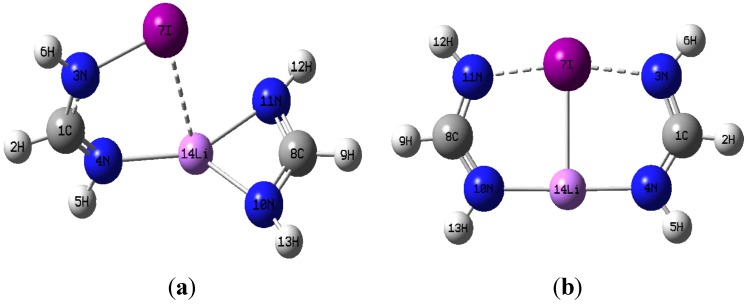
(**a**) optimized dimer geometry of C_1_ symmetry; (**b**) optimized dimer geometry of C_2v_.

M06-2X geometry optimizations resulted in two very distinct dimer structures of C_1_ and C_2v_ symmetry respectively, and they are illustrated in Figure 2a,b using the I-containing dimer as an example.

Table 1 shows selected geometrical parameters obtained with the M06-2X method for the monomers and dimers using the numbering scheme given in Figure 2a,b. With regard to the C_2v_ structures, it is strikingly apparent that dimer formation brings about drastic geometrical distortions in the constituent monomers. One noticeable change is the disappearance of the intramolecular N_10_-Li-N_11_ chelate coordination of the lithium metal in the CH(NH_2_)Li monomer, and the development of quasi-linear N_10_···Li···N_4_ interactions in the dimer with two equal N···Li distances. Accordingly, the N_10_-Li bond is elongated relative to the isolated monomer, the N_11_-Li bond is actually broken completely, and a new N_4_···Li bonding interaction appears upon formation of the C_2v_ dimer. The extent of these changes increases with increasing size of the halogen present in the dimer. For example, the percentage change of the N_10_-Li bond length increases in the order: 2.9% (Cl) < 3.5% (Br) < 4.5% (I).

**Table 1:** Selected geometrical parameters for HN=CHNHX and CH(NH_2_)Li, anddimers at the M06-2X/aug-cc-pVTZ level. Distances in Å, and angles in degrees.

HN=CHNHX						CH(NH_2_)Li				
X	N4-C1	C1-N3	N3-X	τ_XN3H6C1_		N10-C8	C8-N11	N10-Li	N11-Li	
Cl	1.259	1.383	1.702	138.1		1.321	1.321	1.879	1.879	
Br	1.260	1.380	1.848	140.2						
I	1.263	1.373	2.032	145.5						
	Dimers of C_2v_ symmetry									
X	N4-C1	C1-N3	N3-X	N10-C8	C8-N11	N10-Li	N11-Li	N11∙∙∙X	N4∙∙∙Li	Li∙∙∙X
Cl	1.305	1.318	1.964	1.305	1.318	1.933	2.979	1.933	1.964	2.323
Br	1.306	1.320	2.071	1.306	1.320	1.945	3.086	1.945	2.071	2.437
I	1.308	1.323	2.228	1.308	1.323	1.963	3.220	1.963	2.228	2.594
X	Θ_N3XN11_	Θ_N20LiN4_	Θ_XLiN10_	τ_XN3H6C1_						
Cl	175.2	175.8	92.1	180.0						
Br	171.9	178.6	90.7	180.0						
I	166.9	178.7	89.3	180.0						
	Dimer of C_1_ symmetry									
X	N4-C1	C1-N3	N3-X	N10-C8	C8-N11	N10-Li	N11-Li	N11∙∙∙X	N4∙∙∙Li	Li∙∙∙X
Cl	1.270	1.361	1.696	1.318	1.321	1.934	1.964	2.040	3.229	2.606
Br	1.272	1.356	1.839	1.318	1.321	1.934	1.966	2.036	3.368	2.769
I	1.277	1.347	2.025	1.318	1.321	1.940	1.971	2.033	3.530	2.961
X	Θ_N3XN11_	Θ_N10LiN4_	Θ_XLiN10_	τ_XN3H6C1_						
Cl	131.5	134.8	109.0	147.0						
Br	124.5	135.2	107.3	153.1						
I	117.0	134.6	104.7	172.7						

Even more substantial elongations, relative to the halogenated formamidine monomers, occur for the N_3_-X bond upon dimerization. The extent of the elongation decreases with halogen size: 15% (Cl) > 12% (Br) > 10% (I). In all cases, the N_3_-X elongation enables the equal sharing of the halogen atom by two nitrogen atoms, N_3_···X···N_11_, in a quasi-linear arrangement. Another distinct change is seen in the geometry around the nitrogen in the amino NHX group which is trigonal planar in the C_2v_ structure but pyramidal in the isolated monomers. The change in the geometry around the nitrogen in the NHX group is evinced by changes in the dihedral angle τ_XN3H6C1_. This angle is 180° in all C_2v_ dimers in agreement with a trigonal planar geometry, but significantly less in the monomers, consistent with a pyramidal geometry. In addition to the N···Li···N, and N···X···N interactions, a Li···X interaction takes place that appears practically orthogonal to the N···X···N and N···Li···N interactions, with X···Li···N angles close to 90°. Given the drastic geometrical changes occurred upon formation of the C_2v_ structures, the identity of each of the individual monomers can be seen as essentially lost. It is apparent, therefore, that the general C_2v_ structure actually represents a new compound rather than the two original monomers simply engaged in non-covalent intermolecular interactions.

With regard to the dimers of C_1_ symmetry, Table 1 shows that dimerization also brings about important geometrical changes. However these changes are not drastic enough to render the formation of a new compound. Indeed, the structures of both the halogenated formamidine and lithium formamidinate monomers are still clearly distinguishable in the dimer. As seen in Table 1, dimerization results in a sizeable elongation of the imino N_4_-C1 bond, and an equally important shortening of the amino C_1_-N_3_ bond, relative to the isolated halogenated formamidine. A small reduction of the N_3_-X bond lengths is observed for all the dimers, in contrast to the major elongation of this bond seen in the C_2v_ dimers. Moreover, a reduction in the pyramidal character of the amino nitrogen is manifested in a sizeable increase in the τ_XN3H6C1_dihedral angle. The two symmetrical N-Li bonds in the Li-formamidinate monomer are replaced by two somewhat longer and different N-Li bond lengths in the dimer. An additional and important Li…N_4_ interaction takes place with the imino nitrogen of the X-formamidine partner. For any given dimer, the Li…X distance in the C_1_ dimer is significantly longer than that in the corresponding C_2v_ dimer, suggesting a much weaker interaction, if any, between these two atoms in the C_1_ dimer.

Table 2 shows relevant optimized geometrical parameters for the monomers and dimers at the MP2 level of theory. Very interestingly, geometry optimizations with the MP2 method resulted in the C_1_ and C_2v_ dimer structures only for the chlorine-containing dimers. In contrast, the C_2v_ dimer structure was the only one found for the Br- and I-containing dimers. In fact, geometry optimizations for these dimers using the C_1_ structures as initial guess geometry consistently collapsed into the corresponding C_2v_ structures. When compared with the MP2 results, the M06-2X method tends to underestimate bond distances in the monomers and the dimers. For example, cross examination of Table 1 and Table 2 reveals that the M06-2X method predicts N-Li distances for the CH(NH_2_)Li monomer that are 0.038 Å shorter than those predicted by the MP2 method. Also, the hybrid functional predicts a double bond N_4_-C_1_ for the HN=CHNHX monomer that is 0.014 Å shorter than that predicted by the MP2 method. The N-I bond lengths are actually overestimated by the hybrid functional in both monomer and dimers. It can also be seen that the M06-2X method tends to overestimate the τ_XN3H6C1_ dihedral angle for the monomers by about 3° to 4°, and for the C_1_ dimer by about 6°.

### 3.2. Vibrational Frequency Shifts

Dimerization is expected to result in shifts in the vibrational frequencies of the individual monomers, and in the appearance of new modes of vibrations characteristic of the newly formed dimer. Table 3 shows selected harmonic vibrational frequencies of all halogenated formamidine monomers and of their dimers with lithium formamidinate calculated with the M06-2X hybrid functional and the MP2 method using the aug-cc-pVDZ basis set (aug-cc-pVDZ-PP basis set was used for the iodine atom). For the lithium formamidinate monomer, a vibrational stretching frequency involving the chelate N-Li-N moiety, ν_(N-Li-N)_, is found at 575 cm^−1^ with the M06-2X method, and at 550 cm^−1^ with the MP2 method. This mode is still present in the dimers of C_1_ symmetry although it is shifted to the blue, with the largest shift seen in the Cl-containing dimer. The calculated larger frequency for this mode is consistent with the additional interaction of the lithium atom with the imino nitrogen on formamidine. For the Cl-containing dimer, the MP2 method also predicts a blue shift of 39 cm^−1^ for this mode, similar to the 33 cm^−1^ shift seen with M06-2X.

**Table 2:** Selected geometrical parameters for HN=CHNHX and CH(NH_2_)Li, and dimers at the MP2/aug-cc-pVTZ level. Distances in Å, and angles in degrees.

HN=CHNHX						CH(NH_2_)Li				
X	N4-C1	C1-N3	3-X	τ_XN3H6C1_		N10-C8	C8-N11	N10-Li	N11-Li	
Cl	1.273	1.386	1.711	133.8		1.329	1.329	1.916	1.916	
Br	1.274	1.383	1.847	135.8						
I	1.276	1.375	2.022	142.2						
Dimers of C_2v_ symmetry										
X	N4-C1	C1-N3	N3-X	N10-C8	C8-N11	N10-Li	N11-Li	N11∙∙∙X	N4∙∙∙Li	Li∙∙∙X
Cl	1.314	1.324	1.969	1.314	1.324	1.957	3.029	1.969	1.957	2.374
Br	1.315	1.326	2.064	1.315	1.326	1.966	3.123	2.064	1.966	2.473
I	1.317	1.330	2.204	1.317	1.330	1.985	3.250	2.204	1.985	2.619
X	Θ_N3XN11_	Θ_N10LiN4_	Θ_XLiN10_	τ_XN3H6C1_						
Cl	175.8	178.9	90.6	180.0						
Br	173.0	178.7	86.5	180.0						
I	168.5	175.9	87.9	180.0						
Dimer of C_1_ symmetry										
X	N4-C1	C1-N3	N3-X	N10-C8	C8-N11	N10-Li	N11-Li	N11∙∙∙X	N4∙∙∙Li	Li∙∙∙X
Cl	1.282	1.366	1.705	1.327	1.330	1.967	2.000	3.195	2.081	2.618
X	Θ_N3XN11_	Θ_N10LiN4_	Θ_XLiN10_	τ_XNH6C1_						
Cl	134.8	136.5	111.0	140.8						

**Table 3:** Selected stretching (cm^−1^) frequencies for HN=CHNHX and their dimers with CH(NH_2_)Li.

M06-2X/aug-cc-pVDZ							
	HN=CHNHX		Dimer of C_1_ symmetry		Dimers of C_2v_ symmetry		
X	ν_(N-X)_		ν_(N-X)_	ν_as(N-Li-N)_	ν_(N__∙∙∙X__∙∙∙N)_	ν_as(N__∙∙∙Li__∙∙∙N)_	ν_(Li__∙∙∙X)_
Cl	659		688	608	236	663	294
Br	586		607	602	286	640	307
I	540		550	596	327	607	326
**MP2/aug-cc-pVDZ**							
	HN=CHNHX		Dimer of C_1_ symmetry		Dimers of C_2v_ symmetry		
X	ν_(N-X)_		ν_(N-X)_	ν_as(N-Li-N)_	ν_(N__∙∙∙X__∙∙∙N)_	ν_as(N__∙∙∙Li__∙∙∙N)_	ν_(Li__∙∙∙X)_
Cl	637		664	589	304	647	342
Br	576				313	633	338
I	539				337	604	356

As expected, the ν_(N-Li-N)_ mode of vibration is missing in the dimers of C_2v_ symmetry because the intramolecular lithium formamidinate ring is no longer present in these dimers. However, a stretching mode, ν_(N···Li···N)_, appears in the C_2v_ dimers that corresponds primarily with the linear N···Li···N bonds. Both M06-2X and MP2 predict a decrease of the ν_(N···Li···N)_ frequency with an increase in the size of the halogen atom. One distinctive mode of vibration in the halogenated formamidine, ν_N-X_, involves stretching of the N-X bond in the amino group. This mode is shifted to the blue in the dimers of C_1_ symmetry, in accord with the reduction in the corresponding bond lengths discussed in the previous section. In the C_2v_ dimers, however, a vibrational stretching mode encompassing the linear N···X···N interactions, ν_(N···X···N)_, is observed. Because of the relatively large N···X separation, the ν_(N···X···N)_ frequencies appear significantly shifted to the red when compared with the ν_N-X_ frequencies in the corresponding monomers or C_1_ dimers. Lastly, a well defined mode of vibration that emerges upon formation of the C_2v_ structures is a halogen-metal, ν_Li···X_, stretching mode which occurs at low wavenumbers for the various X-containing dimers as seen in Table 3. This mode is clearly absent in the dimers of C_1_ symmetry. It is worth noticing that the ν_(N···X···N)_ and ν_Li···X_ frequencies obtained with M06-2X occur at lower wavenumbers than the corresponding frequencies obtained with the MP2 method. The opposite is true for the calculated ν_(N···Li···N)_ frequencies.

### 3.3. Energetics

Of the two distinct dimer structures obtained with the M06-2X hybrid functional, the one with C_2v_ symmetry is found lower in energy than that of C_1_ symmetry. The energy difference is very small for the Cl-containing dimer, but increases significantly with the size of the halogen. The relative energy results for a given X-containing dimer, E_C1_(X)-E_C2v_(X), using the aug-cc-pVTZ (aug-cc-pVTZ-PP for the iodine atom) follow the sequence: 0.44 kcal/mol (Cl) < 10.1 kcal/mol (Br) < 17.2 kcal/mol (I). The MP2 method favors the C_2v_ dimer more than the M06-2X functional does. In fact at the MP2 level, only the Cl-containing dimer is found to exist as a dimer of C_1_ and C_2v_ symmetry, with the latter being 4.50 kcal/mol lower in energy. For the other two halogens, Br and I, only the C_2v_ dimer structures are predicted to exist.

The BSSE-corrected interaction energies, ΔE^int^, listed in Table 4 were obtained as the difference between the energy of the dimer and the sum of the energies of the constituent monomers, assuming they have the same geometries as in the corresponding dimers. Also listed in Table 4 are the complexation energies, ΔE^c^^omp^, of the dimers which take into account the increase in energy of the monomers due to the geometrical deformations that each monomer goes through upon dimer formation. It is worth noticing that both M06-2X and MP2 yield similar results. With regard to the C_1_ dimers, Table 4 shows that all these dimers have interaction energies (and complexation energies) that are within 1 kcal/mol of one another, although their magnitudes tend to increase slightly with the size of the halogen. Moreover, for any given C_1_ dimer, both the interaction and complexation energies are close to each other, with the largest difference (1.35 kcal/mol) seen for the I-containing dimer. The closeness of the interaction and complexation energies for the C_1_ dimers is consistent with the relatively small geometric deformations that occur during dimer formation. In sharp contrast, all the C_2v_ dimers have interaction energies that are significantly larger than the corresponding complexation energies. The comparatively much smaller complexation energies in the C_2v_ dimers align with the large geometric deformations of the constituent monomers upon C_2v_ dimer formation. The deformation energy varies with the size of the halogen, and for the M06-2X functional the trend is: Cl (42.14 kcal/mol) > Br (38.08 kcal/mol) > I (35.28 kcal/mol), with similar results for the MP2 method. For the C_2v_ dimers, both the interaction and complexation energies display an important increase with the size of the halogen. When comparing the C_1_ and C_2v_ dimer energetics, it is evident that the C_2v_ structures exhibit stronger interactions with the difference being more pronounced the larger the halogen atom is.

**Table 4:** Interaction energies, ΔE^int^, and complexation energies, ΔE^comp^, using the aug-cc-pVTZ basis set. Values in kcal/mol.

			C_2v_-Dimers		
	M06-2X			MP2	
X	ΔE^int^	ΔE^comp^		ΔE^int^	ΔE^comp^
Cl	−62.02	−19.88		−62.77	−22.17
Br	−67.97	−29.88		−66.84	−30.56
I	−72.76	−37.48		−69.32	−36.37
			C_1_-Dimers		
	M06-2X			MP2	
X	ΔE^int^	ΔE^comp^		ΔE^int^	ΔE^comp^
Cl	−20.72	−19.57		−19.79	−18.79
Br	−21.03	−19.83			
I	−21.58	−20.23			

### 3.4. AIM Analysis

**Figure 3:**
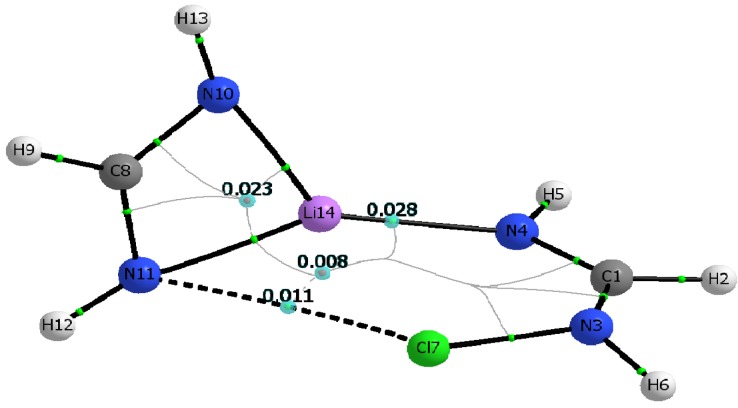
Molecular graph of the C_1_ symmetry dimer of chlorinated formamidine and lithium formamidinate. Bond critical points, ring critical points, and bond paths are shown. Molecular graph obtained using B3LYP/aug-cc-pvtz wavefunction obtained from the M06-2X/aug-cc-pvtz optimized dimer.

Additional insight on the nature and strength of the various N···Li, N···X, and X···Li interactions is gained by analyzing relevant topological parameters of the electron densities at the corresponding critical points [46]. The calculated molecular graphs for all C_1_ dimers mirror that of the Cl-containing dimer shown in Figure 3. Table 5 lists the electron densities at the critical points of the intermolecular N···Li and N···X interactions, ρ_c_(N_4_···Li), and ρ_c_(N_11_···X), as well as their corresponding total electron energy densities, H_c_(N_4_···Li), and H_c_(N_11_···X), calculated for both the M06-2X and MP2 geometries. Also listed in Table 5 are the electron densities at the two ring critical points found in the dimer. Inspection of Figure 3 and Table 5 makes readily apparent that the structural identity of the constituent monomers remains unaltered upon formation of the C_1_ dimer. For example, the ring critical point of the Li-formamidinate unit is still present in the dimer, and the geometry around the amino nitrogen remains pyramidal. Formation of the C_1_ dimer is driven mostly by the N···Li interaction and to a lesser extent by the N···X interaction. Thus, the C_1_ dimers exhibit ρ_c_(N···Li) values that are about three times larger than the ρ_c_(N···X) values. Similarly, the electron density value of the ring critical point of the formamidinate unit is about three times larger than that of the six-membered ring resulting from formation of a C_1_ dimer. The positive signs of the total energy densities, H_c_(N···Li) and H_c_(N···X), confirm the non-covalent nature of these interactions [47]. The presence of a relatively weak halogen bond, N···X, as established by the existence of its bond critical point is particularly noteworthy given the unfavorable interaction angles Θ_N3-X···N11_. Indeed, for any given C_1_ dimer, Θ_N3‑X···N11_ is severely deviated from linearity, and it gets even smaller as the halogen gets bigger: 131.5° (Cl) > 124.5° (Br) > 117.0° (I). Yet another striking feature of the C_1_ dimers is the absence of a critical point linking the lithium and the halogen atom. 

**Table 5:** Intermolecular bond and ring critical point electron densities (ρ, au), and bond energy densities (H, au) in the dimers of C_1_ symmetry*.

M06-2X				
ρ_c (N∙∙∙X)_	ρ_c (N∙∙∙Li)_	H_c (N∙∙∙X)_	H_c (N∙∙∙Li)_	ρ_rcp1_
0.0107	0.0275	0.0012	0.0058	0.0228
0.0102	0.0278	0.0009	0.0058	0.0227
0.0100	0.0281	0.0007	0.0058	0.0225
MP2				
ρ_c (N∙∙∙X)_	ρ_c (N∙∙∙Li)_	H_c (N∙∙∙X)_	H_c (N∙∙∙Li)_	ρ_rcp1_
0.0112	0.0248	0.0012	0.0057	0.0210

* Properties calculated with the B3LYP functional on the M06-2X and MP2 geometries, rcp1 refers to the 4-membered ring, and rcp2 refers to the 6-membered ring in Figure 3.

**Figure 4:**
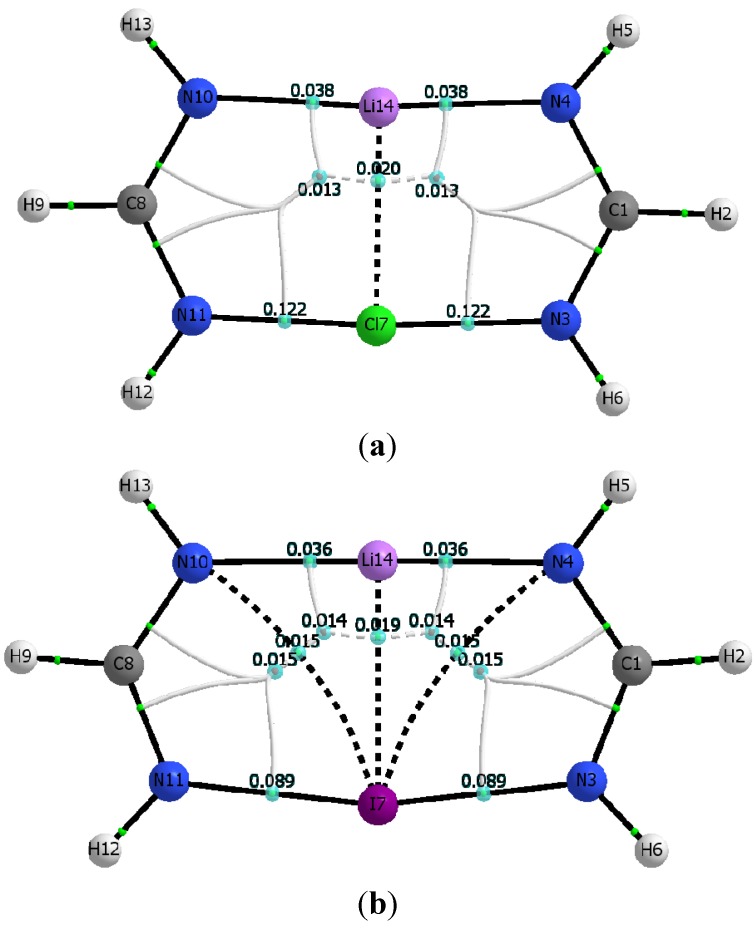
Molecular graph of dimers with C_2v_ symmetry (**a**) dimer of chlorinated formamidine and lithium formamidinate. (**b**) dimer of iodinated formamidine and lithium formamidinate. Bond critical points, ring critical points, and bond paths are sown. Molecular graphs obtained using B3LYP/aug-cc-pvtz wavefunction obtained from the M06-2X/aug-cc-pvtz optimized dimers.

The calculated molecular graphs for the Cl- and Br-containing dimers of C_2v_ symmetry look alike, and in particular the molecular graph of the former is shown in Figure 4a. Similarly, Figure 4b shows the molecular graph calculated for the I-containing C_2v_ dimer.

Figure 4a,b consistently show that all C_2v_ dimers have a set of parallel and quasi-linear N_4_···Li···N_10_, and N_11_···X···N_3_, interactions that are connected by orthogonal Li…X interactions. For X = I, however, Figure 4b shows two additional curved bond paths connecting the iodine atom with the two farther nitrogen atoms, N_4_ and N_10_ respectively. Because of these additional interactions, the I-containing C_2v_ dimer has two equivalent 4-membered rings and two equivalent 3-membered rings, rather than the two symmetric 5-membered rings seen when the C_2v_ dimer contains either of the other two halogen atoms, Br or Cl. Table 6 lists electron densities at the relevant intermolecular bond critical points, as well as their corresponding total electron energy densities, calculated for both the M06-2X and MP2 geometries. Also listed in Table 6 are the electron densities at the ring critical points found in each of the C_2v_ dimers.

Inspection of Figure 4a,b and Table 6 demonstrates that the structural identity of each constituent monomer in the C_2v_ dimers is drastically altered. Particularly outstanding is the disappearance of one of the N-Li bond critical points present in the Li-formamidinate monomer, and hence the disappearance of the formamidinate ring unit as such. Instead, a well-defined Li···X bond critical point is present accompanied by two equivalent bond critical points in quasi-linear N…X…N interactions (as opposed to the severely curved N-X···N interactions seen in the C_1_ dimers). Formation of the C_2v_ dimer appears then driven by the interplay of at least three major interactions: quasi-linear and parallel N_4_···Li···N_10_ and N_11_···X···N_3_ interactions, with orthogonal Li···X interactions.

As seen in Table 6, the quasi-linear interactions are characterized by electron density values at their critical points that are significantly larger than the corresponding values in the C_1_ dimer counterparts (see Table 5). Another salient finding is that, for any given C_2v_ dimer, the N_11_···X···N_3_ interactions appear stronger than the adjacent and parallel N_4_···Li···N_10_ interactions. Indeed, ρ_c(N11···X···N3)_ values are larger by a factor of about 3 in the Br- and Cl-containing dimers, and by a factor of 2.5 in the I-containing dimer. The two additional N_4_···I and N_10_···I interactions seen in the I-containing dimer provide further stabilization and help drive the formation of the C_2v_ dimer. It is worth noticing that although the electron density values at the bond critical points of these additional interactions are smaller than those of their quasi-linear counterparts in the C_2v_ dimer, they are actually larger than that of the intermolecular N···I interaction in the C_1_ dimer. As in the C_1_ dimers, it is important to stress that the presence of the relatively weak and curved halogen bonds, N_4_···I and N_10_···I as confirmed by the existence of their bond critical point is remarkable given the unfavorable interaction angles. For example, Θ_N3···X···N4_ is about 120°, just a bit larger than Θ_N3-X···N11_ for the halogen bond in the C_1_ dimer. Lastly, it is equally important to highlight the fact that the quasi-linear halogen bonds N_11_···X···N_3_ exhibit a partly covalent nature, as revealed by the negative sign of the total electronic energy densities, evaluated at the pertinent bond critical points, H_c_. In contrast, the weaker N_4_···I (or N_10_…I) halogen bond interaction, the Li···X, and the N_4_···Li···N_10_ interactions all exhibit a non-covalent closed-shell interaction (all with H_c_ > 0) [47,48,49].

**Table 6:** Intermolecular bond and ring critical point electron densities (ρ, au), and bond energy densities (H, au) in the dimers of C_2v_ symmetry *.

	M06-2x							
X	ρ_c (N__∙∙∙X)_	ρ_c (N__∙∙∙Li)_	ρ_c (Li__∙∙∙X)_	H­_c (N__∙∙∙X)_	H_c (N__∙∙∙Li)_	H_c (Li__∙∙∙X)_	ρ_rcp1_	ρ_rcp2_
Cl	0.1222	0.0377	0.0202	−0.0500	0.0054	0.0046	0.0133	0.0133
Br	0.1066	0.0351	0.0197	−0.0472	0.0052	0.0036	0.0139	0.0139
I	0.0888	0.0355	0.0188	−0.0346	0.0052	0.0026	0.0145	0.0145
	0.0150			0.0008			0.0143	0.0143
	MP2							
X	ρ_c (N__∙∙∙X)_	ρ_c (N__∙∙∙Li)_	ρ_c (Li__∙∙∙X)_	H_c (N__∙∙∙X)_	H_c (N__∙∙∙Li)_	H_c (Li__∙∙∙X)_	ρ_rcp1_	ρ_rcp2_
Cl	0.1211	0.0358	0.0177	−0.0471	0.0052	0.0044	0.0123	0.0123
Br	0.1084	0.0351	0.0180	−0.0460	0.0052	0.0034	0.0132	0.0132
I	0.0926	0.0336	0.0178	−0.0346	0.0052	0.0025	0.0145	0.0145
	0.0149			0.0008			0.0139	0.0139

* Properties calculated with the B3LYP functional on the M06-2X and MP2 geometries rcp1 and rcp2 refers to the symmetric 5-membered rings for X = Cl or Br (Figure 4a); and to the 4- and 3-membered rings respectively for X = I (Figure 4b).

### 3.5. Polymer Synthons

**Figure 5:**
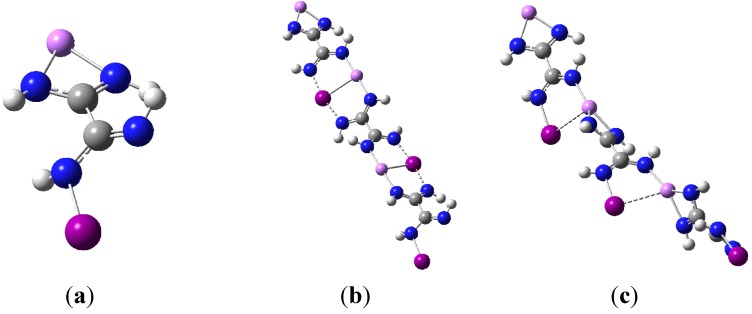
(**a**) a simple hypothetical polymeric synthon containing both the lithium formamidinate and the iodinated formamidine moieties; (**b**) a trimer built sequentially by using the simple polymeric synthon of Figure 5a through the combination of partly covalent N···I···N halogen bonding, closed-shell N···Li···N, and orthogonal Li···I interactions; (**c**) a trimer built sequentially by using the simple polymeric synthon of Figure 5a through purely non-covalent interactions.

Given the robustness of the interactions found for all dimers considered in this study, it is tempting to envision a molecular motif possessing both the lithium formamidinate and the halogenated formamidine moieties separated by a group of atoms, R, within the same molecule, *i.e.*, Li(NH)_2_-R-(NH)_2_X. Such motifs may find application, for example, as polymeric synthons. As a straightforward example, the monomeric structure shown in Figure 5a was used as a convenient synthon to build sequentially dimer and trimer structures. For each dimer and trimer, two possible structures were considered: One based on the non-covalent interactions of the interacting monomers, so that the identity of monomers remains unaltered (similar to the C_1_ dimers discussed above); the other based on the partly-covalent halogen bond interactions (again, similar to the C_2v_ dimers discussed above). For all these calculations, the M06-2X/aug-cc-pVDZ method was used (with aug-cc-pVDZ-PP used for the iodine atom). In particular, the optimized geometries of the two trimers are shown in Figure 5b–c. The BSSE-corrected complexation energy of forming either the dimer or the trimer based on the partly covalent halogen bonds is about ‒36 kcal/mol. The corresponding interaction energy is about ‒68 kcal/mol. For the dimers based on the closed-shell interactions, the complexation energies are about ‒23 kcal/mol with corresponding interaction energies that are within ‒1 kcal/mol. Overall, these values compare well with those obtained for the lithium formamidinate and iodinated formamidine dimers discussed earlier (see Table 4).

## 4. Conclusions

The optimized geometries for the dimer interaction between lithium formamidinate and halogenated formamidines HN=CHNHX, (X = Cl, Br, or I), were examined using the MP2 method and the M06-2X hybrid functional. Both MP2 and M06-2X predict the formation of a very stable planar structure of symmetry C_2v_ regardless of the identity of the halogen atom. In this structure, the identities of the constituent monomers are essentially lost. Upon formation of the C_2v_ dimers, one of the N-Li bonds in lithium formamidinate is completely broken, and therefore the ring structure of this monomer is no longer present in the dimer. Formation of the C_2v_ structure occurs also with a quasi-linear N···Li···N interaction parallel to the N···X···N interaction. Each of the N···Li interactions is found to be non-covalent in nature. Furthermore, a non-covalent Li···X interaction is found orthogonal to both the N···X···N and N···Li···N interactions. The strength of the interactions increases with the size of the halogen. The M06-2X hybrid functional also predicts a dimer structure of C_1_ symmetry for all halogens that are higher in energy than their C_2v_ counterparts. In the C_1_ dimers, the identity of each unit remains unchanged and dimer formation is driven by intermolecular N···Li, and N···X closed-shell interactions. At the MP2 level, the C_1_ dimer is predicted to exist only for X=Cl. For the other two halogens, the C_1_ structures do not exist, but rather they collapse into the more stable C_2v_ structures. The robustness of the interactions driving the formation of the dimer structures suggests that suitable analogues of the systems studied here may find diverse applications including their use as novel polymeric synthons [50,51]. Although some illustrative results in this regard were presented here, it certainly remains a subject of future work. It is also of interest to investigate whether similarly strong interactions X···N, or X···O can be found with other halogen containing motifs such as R-O-X, and R-X. Efforts in these directions are currently underway in the author’s lab.

## Supplementary Materials

Supplementary materials can be accessed at: http://www.mdpi.com1420-3049/19/1/1069/s1.
