# Supplementary material for: Concerted Halogen Bonding and Orthogonal Metal-Halogen Interactions in Dimers of Lithium Formamidinate and Halogenated Formamidines: An ab Initio Study
Source: Molecules. 2014 Jan 17;19(1):1069–84. doi: 10.3390/molecules19011069 (PMC6271871; doi:10.3390/molecules19011069)
Supplement: Supplementary file 1 [file molecules-19-01069-s001.pdf]

Supplementary Materials

MP2/aug-cc-pvtz Geometries and Energies

Monomers

Lithium Formamidinate
Energy(au): -156.66009280

```
0 1
 C              -0.00000200   -0.58212200    0.00001200
 N              -1.14439300    0.09326800    0.00002900
 N               1.14439300    0.09326500   -0.00003700
 H               1.94970400   -0.51347000    0.00006600
 H              -1.94971000   -0.51345800    0.00017100
 Li              0.00000700    1.63048400   -0.00012200
 H              -0.00000200   -1.67752700    0.00011100
```

Iodinated Formamidine
Energy(au): -444.02502051

```
0 1
 C               2.12230400    0.33086200    0.03018300
 H               2.95611900    1.02924500    0.12874800
 N               2.21307600   -0.94168500   -0.01213600
 N               0.92512100    0.99179200   -0.11767900
 H               0.85853400    1.89975700    0.31811600
 H               3.18843400   -1.22357600    0.03344100
 I              -0.78687300   -0.07625200    0.00466600
```

Brominated Formamidine
Energy(au): -2721.80027743

```
0 1
 C               1.76063200    0.36883800    0.03096600
 H               2.53779600    1.12885400    0.13620800
 N               1.94052900   -0.89170400   -0.01331100
 N               0.52195800    0.96047700   -0.13503600
 H               0.38162500    1.81863900    0.37991800
 H               2.93417100   -1.09784200    0.04620100
 Br             -0.96156500   -0.12983100    0.00829400
```

Chlorinated Formamidine
Energy(au): -608.80594264

```
0 1
 C               1.24091900    0.41918500    0.02716500
 H               1.92418000    1.26563500    0.12213000
 N               1.56071500   -0.81248300   -0.01069400
 N              -0.05929600    0.87140900   -0.13656500
 H              -0.29289400    1.69507300    0.40122800
 H               2.57156100   -0.90240000    0.04887700
 Cl             -1.30342900   -0.29328800    0.01738800
```

MP2/aug-cc-pvtz Geometries and Energies
Dimers of C_{2v} symmetry

I-containing C_{2v}-dimer
Energy(au): -600.75452839

0 1

C	0.00000000	2.70590000	-0.73501900
H	0.00000000	3.79649100	-0.79737000
N	0.00000000	2.19296400	0.49162500
N	0.00000000	1.98361300	-1.83588000
H	0.00000000	2.61207200	-2.63120100
H	0.00000000	2.86772000	1.24203400
C	0.00000000	-2.70590000	-0.73501900
H	0.00000000	-3.79649100	-0.79737000
N	0.00000000	-1.98361300	-1.83588000
N	0.00000000	-2.19296400	0.49162500
H	0.00000000	-2.86772000	1.24203400
H	0.00000000	-2.61207200	-2.63120100
I	0.00000000	0.00000000	0.71195600
Li	0.00000000	0.00000000	-1.90693400

Br-containing C_{2v}-dimer
Energy (au): -2878.51644845

0 1

C	0.00000000	2.63718600	-0.52174900
H	0.00000000	3.72977600	-0.52476100
N	0.00000000	2.06003500	0.67231800
N	0.00000000	1.96603900	-1.65246100
H	0.00000000	2.62051500	-2.42538000
H	0.00000000	2.66683600	1.47828100
C	0.00000000	-2.63718600	-0.52174900
H	0.00000000	-3.72977600	-0.52476100
N	0.00000000	-1.96603900	-1.65246100
N	0.00000000	-2.06003500	0.67231800
H	0.00000000	-2.66683600	1.47828100
H	0.00000000	-2.62051500	-2.42538000
Li	0.00000000	0.00000000	-1.67453200
Br	0.00000000	0.00000000	0.79858000

Cl-containing C_{2v}-dimer
Energy (au): -765.50504603

0 1

C	2.58910600	0.27634100	-0.00016500
H	3.68122900	0.23557300	-0.00115500
N	1.96777100	-0.89324200	0.00042600
N	1.95735600	1.42828400	0.00043300
H	2.63295700	2.18201800	-0.00002200
H	2.52423700	-1.73480200	-0.00070800
C	-2.58910700	0.27633500	0.00040300
H	-3.68123000	0.23556400	0.00187800
N	-1.95736200	1.42828200	-0.00049700
N	-1.96776900	-0.89324500	-0.00050400
H	-2.52423000	-1.73480700	0.00127800
H	-2.63297100	2.18201000	0.00019300
Li	-0.00000800	1.40927500	-0.00022700

Cl	0.00000400	-0.96470600	-0.00007100

MP2/aug-cc-pvtz Geometries and Energies
Dimers of C_1 symmetry

Cl-containing C_1-dimer
Energy(au): -765.49788239

0 1

C	-2.27428800	0.88580600	0.08135400
H	-3.28215400	1.29499200	0.03489100
N	-2.22417500	-0.39710800	-0.38573600
N	-1.23464300	1.53261700	0.46129700
H	-1.48664000	2.48862700	0.69137200
H	-3.04482500	-0.97252600	-0.26722600
C	2.78786900	-0.01987200	-0.11313400
H	3.76477500	-0.43824500	-0.38694500
N	2.24157700	0.94450700	-0.84240200
N	2.13442700	-0.47750700	0.95083300
H	2.66565200	-1.18910900	1.43282800
H	2.82652500	1.19697500	-1.62585200
Li	0.72326300	0.83034400	0.40225700
Cl	-0.77146500	-1.25205400	-0.12841800

M062X/aug-cc-pvtz Geometries and Energies
Monomers

Lithium Formamidinate
Energy(au): -156.97763266

0 1

C	0.00000000	0.57241000	0.00011500
N	1.13951000	-0.09548800	-0.00001700
N	-1.13951000	-0.09548800	-0.00007900
H	-1.95032400	0.50042600	-0.00007700
H	1.95032400	0.50042600	0.00003100
Li	0.00000000	-1.58918700	-0.00005900
H	0.00000000	1.66908700	0.00020400

Iodinated Formamidine
Energy(au): -444.99819851

0 1

C	2.13745300	0.32815900	0.02916100
H	2.96104100	1.04026000	0.13558000
N	2.26087700	-0.92791700	-0.01396600
N	0.93587700	0.97697600	-0.11153700
H	0.86987700	1.90119900	0.28352800
H	3.23519600	-1.20206500	0.03881100
I	-0.79751200	-0.07644800	0.00463500

Brominated Formamidine
Energy(au): -2723.60472537

```
0 1
 C                1.76967600    0.36479400    0.02657700
 H                2.53798200    1.13679700    0.12477300
 N                1.97375400   -0.87808600   -0.01102400
 N                0.52652300    0.94400000   -0.12245300
 H                0.38658700    1.82814000    0.34102200
 H                2.96500600   -1.08438400    0.04401300
 Br              -0.97170200   -0.12944900    0.00757300
```

Chlorinated Formamidine
Energy(au): -609.56236394

```
0 1
 C                1.24221700    0.41401900    0.02196900
 H                1.91942000    1.26798000    0.10632200
 N                1.57767300   -0.79939800   -0.00743200
 N               -0.06069300    0.85522600   -0.12307400
 H               -0.29753500    1.70541700    0.36557800
 H                2.58596700   -0.89443900    0.04537600
 Cl              -1.31058900   -0.29140400    0.01555600
```

M062X/aug-cc-pvtz Geometries and Energies
Dimers of C_{2v} symmetry

I-containing C_{2v}-dimer
Energy(au): -602.03600070

```
0 1
 C                0.00000000    2.69454300   -0.76041700
 H                0.00000000    3.78491700   -0.84581300
 N                0.00000000    2.21329700    0.47185000
 N                0.00000000    1.96246200   -1.84411700
 H                0.00000000    2.56407100   -2.65665100
 H                0.00000000    2.89900000    1.20927700
 C                0.00000000   -2.69454300   -0.76041700
 H                0.00000000   -3.78491700   -0.84581300
 N                0.00000000   -1.96246200   -1.84411700
 N                0.00000000   -2.21329700    0.47185000
 H                0.00000000   -2.89900000    1.20927700
 H                0.00000000   -2.56407100   -2.65665100
 Li               0.00000000    0.00000000   -1.86677800
 I                0.00000000    0.00000000    0.72685800
```

Br-containing C_{2v}-dimer
Energy (au): -2880.63075898

```
0 1
 C                0.00000000    2.62027700   -0.53582200
 H                0.00000000    3.71390700   -0.55512700
 N                0.00000000    2.06619100    0.66231500
 N                0.00000000    1.94526900   -1.65398300
 H                0.00000000    2.57947100   -2.44037200
 H                0.00000000    2.68399100    1.45707800
 C                0.00000000   -2.62027700   -0.53582200
 H                0.00000000   -3.71390700   -0.55512700
 N                0.00000000   -1.94526900   -1.65398300
 N                0.00000000   -2.06619100    0.66231500
 H                0.00000000   -2.68399100    1.45707800
```

H	0.00000000	-2.57947100	-2.44037200
Li	0.00000000	0.00000000	-1.62944600
Br	0.00000000	0.00000000	0.80795400

Cl-containing C_{2v}-dimer
Energy (au): -766.57253841

0 1

C	0.00000000	2.56642000	-0.28436900
H	0.00000000	3.66012100	-0.25824200
N	0.00000000	1.96253400	0.88706100
N	0.00000000	1.93169900	-1.42475700
H	0.00000000	2.58704000	-2.19287600
H	0.00000000	2.53019100	1.71829500
C	0.00000000	-2.56642000	-0.28436900
H	0.00000000	-3.66012100	-0.25824200
N	0.00000000	-1.93169900	-1.42475700
N	0.00000000	-1.96253400	0.88706100
H	0.00000000	-2.53019100	1.71829500
H	0.00000000	-2.58704000	-2.19287600
Li	0.00000000	0.00000000	-1.35465700
Cl	0.00000000	0.00000000	0.96881100

M062X/aug-cc-pvtz Geometries and Energies
Dimers of C_1 symmetry

I-containing C_1-dimer
Energy(au): -602.00860190

0 1

C	1.52282300	2.00238100	-0.06119300
H	2.30724500	2.75513500	0.02857400
N	1.96549000	0.75794300	0.20542400
N	0.31723200	2.28244000	-0.37401900
H	0.20800700	3.27976600	-0.51248400
H	2.94093200	0.60186000	0.38588600
C	-3.16967700	-0.12318300	0.12159600
H	-4.05542000	-0.73103200	0.35186400
N	-2.70886400	0.73922100	1.00533700
N	-2.55795000	-0.25364100	-1.04222600
H	-3.01959800	-0.91438500	-1.64742600
H	-3.24983400	0.74876600	1.85530700
Li	-1.38196400	1.16616600	-0.34448200
I	0.75064800	-0.85274600	0.03108900

Br-containing C_1-dimer
Energy (au): -2880.61458787

0 1

C	-1.89540200	1.49845000	0.07942200
H	-2.81195300	2.08689100	0.02893400
N	-2.10174900	0.20886100	-0.28376100
N	-0.75971800	1.97333600	0.40148800
H	-0.82260200	2.96799100	0.58096400
H	-3.03425700	-0.16743200	-0.26748000
C	2.97842100	-0.01190200	-0.11157500
H	3.93072900	-0.50910300	-0.34205300

N	2.45688500	0.85055100	-0.96111100
N	2.34905200	-0.27584800	1.01953600
H	2.85684100	-0.92509200	1.59945000
H	3.01923600	0.96826400	-1.78854800
Li	1.04991700	1.04102000	0.35159400
Br	-0.75420400	-1.02177600	-0.05446300

Cl-containing C_1-dimer
Energy (au): -766.57183951

0 1

C	-2.22173300	0.90599900	0.07717100
H	-3.22301300	1.33183800	0.01498900
N	-2.19422800	-0.38498200	-0.35329700
N	-1.18799500	1.53996800	0.45462300
H	-1.40893700	2.50390700	0.67238400
H	-3.03551600	-0.93635300	-0.30600700
C	2.74287300	-0.00673100	-0.12201600
H	3.73011000	-0.40726700	-0.39079800
N	2.15253400	0.87929800	-0.89925700
N	2.13973500	-0.41322700	0.98070100
H	2.69599500	-1.07198000	1.50237000
H	2.70044400	1.10607900	-1.71372100
Li	0.71362500	0.80334300	0.39199900
Cl	-0.77041900	-1.27525100	-0.11562000

M062X/aug-cc-pvdz Geometries and Energies
Monomeric synthon

I-containing Synthon:
Energy(au): -600.72102433

0 1

C	-1.12995600	0.42095700	0.22165100
N	-0.81315300	1.61043000	0.54370700
N	-0.18791900	-0.58902600	0.09690000
H	-0.43809100	-1.31112700	-0.57077500
H	-1.65626000	2.16692600	0.68626400
C	-2.55283000	-0.07914400	-0.00578100
N	-2.92743400	-1.29596500	0.37198300
N	-3.44460100	0.70359900	-0.59639700
H	-3.05623700	1.57407000	-0.93654100
H	-2.22353400	-1.78287800	0.91170400
Li	-4.62616100	-0.75402800	-0.29257900
I	1.79171800	-0.06488800	-0.06455600

M062X/aug-cc-pvdz Geometries and Energies
Dimer and trimer based on C_{2v} symmetry

I-containing Synthon: Dimer based on C_{2v}-symmetry
Energy(au): -1201.50206329

0 1

C	-4.33608600	0.69180100	0.09057400
N	-4.04417700	-0.56609100	-0.24018500
N	-3.43647500	1.56406700	0.48543600
H	-3.90946100	2.42872600	0.73571100
H	-4.81018800	-1.10208700	-0.62817500
C	0.99369600	-0.27694800	0.38211900
N	0.42482900	0.87095700	0.68839600
N	0.32922800	-1.34858500	-0.03084100
H	0.90562400	-2.13213900	-0.31220800
H	1.13069400	1.51457800	1.03459800
I	-1.92552700	-1.18868400	-0.21531900
Li	-1.50354700	1.25772800	0.62047500
C	2.50792900	-0.43556400	0.49932300
N	3.19064900	0.68747800	0.05704600
N	3.10517500	-1.44188000	0.99941300
H	2.42595700	-2.10678100	1.36925000
H	2.74418200	1.17575900	-0.71255100
I	5.22529200	0.55884600	-0.19561900
C	-5.80598400	1.09055800	-0.00415200
N	-6.76776400	0.25962500	0.38083200
N	-6.15431000	2.28157800	-0.47571700
H	-6.41778900	-0.57573500	0.83231600
Li	-7.91974600	1.67575500	-0.13763900
H	-5.37411200	2.80954100	-0.84557300

I-containing Synthon: Trimer based on C_{2v}-symmetry
Energy(au): -1802.28314855

0 1

C	-7.88534300	0.08378500	0.17219600
N	-7.37291400	-1.08676500	-0.20603600
N	-7.15291400	1.10543200	0.55654400
H	-7.77113100	1.85882900	0.84637200
H	-8.04053900	-1.75450700	-0.57115300
C	-2.45579000	0.14895100	0.22094900
N	-3.22200900	1.15103500	0.59970100
N	-2.92463700	-1.02860700	-0.17857700
H	-2.22843100	-1.66699400	-0.54389100
H	-2.63678700	1.91358700	0.92988500
I	-5.16813700	-1.29192000	-0.27466800
Li	-5.19036600	1.15760700	0.61938100
C	-0.93633300	0.30638600	0.23548900
N	-0.45883400	1.43917900	-0.27786200
N	-0.17412700	-0.63684500	0.74237800
H	-0.76495800	-1.36663000	1.13218000
H	-1.13911600	2.03274100	-0.73559300
C	4.49069300	0.46855200	0.36354800
N	3.76109400	-0.52395700	0.82821800
N	3.98593300	1.57038500	-0.17901800
H	4.66366400	2.20870400	-0.57712300
H	4.37049300	-1.22150800	1.24611400
I	1.73850200	1.72338300	-0.33680300
Li	1.79266400	-0.61180300	0.83414900
C	6.01399800	0.39833300	0.43943000
N	6.77705900	1.35233100	0.79546200
N	6.49585100	-0.86710100	0.14451100
H	6.22353600	2.16000300	1.08109400
H	5.95785100	-1.37459900	-0.55015800
I	8.51690900	-1.11645100	-0.11595100
C	-9.40694000	0.19440400	0.14947200
N	-10.17238400	-0.80830800	0.56556900
N	-9.99917500	1.29968400	-0.28633100
H	-9.64636300	-1.56506300	0.98375200
Li	-11.59705200	0.36783000	0.13740000
H	-9.35179900	1.97117000	-0.67930700

M062X/aug-cc-pvdz Geometries and Energies
Dimer and trimer based on C_1 symmetry

I-containing Synthon: Dimer based on C_1-symmetry
Energy(au): -1201.47915346

```
0 1
 C                 -2.59272100    1.12974000    0.42612300
 N                 -3.26075100    1.74652100    1.31719100
 N                 -3.20711400    0.37005300   -0.56011700
 H                 -2.64221300   -0.40053700   -0.90142800
 H                 -2.63133200    2.32624800    1.87285100
 C                 -1.07287900    1.17118100    0.26529000
 N                 -0.50880600    1.15187300   -0.93438400
 N                 -0.29893000    1.21374200    1.33830300
 H                 -0.81877800    1.13735700    2.20431300
 H                 -1.18129000    1.23450200   -1.68696300
 Li                 1.19275400    1.27025700    0.04223100
 C                  4.14118600    0.58240800   -0.22153600
 N                  3.18393700    1.43751400   -0.19913600
 N                  3.94437300   -0.76348700   -0.14227800
 H                  4.73779100   -1.32278200    0.14603400
 H                  3.55857600    2.36971200   -0.36929500
 C                  5.60760500    0.96473400   -0.39051900
 N                  6.42617000    0.22651900   -1.12815000
 N                  6.08405800    2.04629500    0.20351300
 H                  5.43147100    2.49410800    0.83449400
 H                  5.95830800   -0.49955600   -1.65587500
 Li                 7.71037000    1.57629200   -0.68523400
 I                  2.10876500   -1.53428800    0.33036500
 I                 -5.16841800   -0.18213200   -0.29745700
```

I-containing Synthon: Trimer based on C_1-symmetry
Energy(au): -1802.24010268

```
0 1
 C                  0.68151500    0.19408700   -0.29012800
 N                 -0.21810400    0.92190900    0.26589100
 N                  0.39512400   -0.96619100   -0.95121700
 H                  1.16668100   -1.61173600   -1.06935400
 H                  0.21307400    1.77842600    0.61117700
 C                  2.16646600    0.55031400   -0.31923100
 N                  2.90105400    0.31196600   -1.39412900
 N                  2.73042500    1.09159400    0.74644100
 H                  2.11388200    1.14369200    1.54871400
 H                  2.35611500    0.02286700   -2.19763600
 Li                 4.37636400    1.13673500   -0.37012800
 C                  7.40971100    1.09488300   -0.34247600
 N                  6.29637200    1.68103900   -0.59825900
 N                  7.49198700   -0.15080100    0.20484600
 H                  8.36983300   -0.39120000    0.64942600
 H                  6.48049400    2.55670700   -1.08588100
 C                  8.77168100    1.69932700   -0.66485100
 N                  9.75849800    0.94914200   -1.13487600
 N                  8.98524600    2.99055000   -0.47441700
 H                  8.22572500    3.47376100   -0.01148600
 H                  9.47844700    0.00752200   -1.37861900
 Li                10.71787600    2.60589400   -1.19647200
 I                  5.83701600   -1.05443800    1.00473700
 I                 -1.44555600   -1.84548200   -0.76065400
```

C	-5.98597800	0.84624000	0.95525100
N	-6.56521800	0.94582600	2.08468000
N	-6.69606800	0.85233600	-0.23729600
H	-6.25666600	0.32666400	-0.98581100
H	-5.86180100	1.03000800	2.81893200
C	-4.47476900	0.77408600	0.73410700
N	-3.90685200	1.36109100	-0.30964500
N	-3.71536900	0.11297700	1.59319000
H	-4.25070900	-0.36467400	2.30816800
H	-4.55338200	1.93205000	-0.84018300
Li	-2.20863500	0.74806300	0.47377100
I	-8.72636500	0.54067700	-0.17033700
